# A Sub-Group of Kidney-Transplant Recipients with Highly Aggressive Squamous Cell Carcinoma Expressing Phosphorylated Serine392p53

**DOI:** 10.3390/ijms25021147

**Published:** 2024-01-17

**Authors:** Diaddin Hamdan, Charlotte Gardair, Frédéric Pamoukdjian, Marie-Noëlle Peraldi Gardin, Inès Nakouri, Christophe Leboeuf, Anne Janin, Céleste Lebbé, Maxime Battistella, Guilhem Bousquet

**Affiliations:** 1 Faculté de Santé, Site Lariboisière, Institut National de la Santé et de la Recherche Médicale INSERM, Unité Mixte de Recherche UMR_S942 MASCOT, Université Paris-Cité, F-75006 Paris, France; 2 Medical Oncology Department, Hôpital La Porte Verte, F-78000 Versailles, France; 3 UFR Santé Médecine et Biologie Humaine, Campus Bobigny, Université Sorbonne Paris Nord, F-93439 Villetaneuse, France; 4 Geriatric Medicine Department, Assistance Publique-Hôpitaux de Paris, Hôpital Avicenne, F-93000 Bobigny, France; 5 Nephrology-Renal Transplantation Department, Assistance Publique-Hôpitaux de Paris, Hôpital Necker-Enfants Malades, F-75015 Paris, France; 6 Faculté de Santé, Assistance Publique-Hôpitaux de Paris Dermato-Oncology, Cancer Institute APHP. Nord Paris Cité, Institut National de la Santé et de la Recherche Médicale INSERM Unité U976, Saint Louis Hospital, Université Paris-Cite, F-75010 Paris, France; 7 Department of Pathology, Assistance Publique-Hôpitaux de Paris, Hôpital Saint-Louis, Faculté de Santé, Cancer Institute APHP. Nord Paris Cité, Institut National de la Santé et de la Recherche Médicale INSERM Unité U976, Université Paris-Cite, F-75010 Paris, France; 8 Medical Oncology Department, Assistance Publique-Hôpitaux de Paris, Hôpital Avicenne, F-93000 Bobigny, France

**Keywords:** kidney transplantation, squamous cell carcinoma, p53 protein, phosphorylation

## Abstract

Cutaneous squamous cell carcinomas in kidney-transplant recipients are frequent, with an increasing incidence linked to long immunosuppression durations and exposure to ultraviolet radiation. p53 is at the cornerstone of ultraviolet-induced DNA damage, but the role of p53 post-translational modifications in this context is not yet deciphered. Here, we investigated the phosphorylation status of p53 at Serine 392 in 25 cutaneous squamous cell carcinomas in kidney-transplant recipients, compared with 22 non-transplanted patients. Cutaneous squamous cell carcinomas in transplanted patients occurred after a median period of 19 years of immunosuppression, with a median number of 15 cutaneous squamous cell carcinomas and more aggressive histological and clinical characteristics. There was no significant difference between Ki67, p53, and pSer392p53 expression in the two groups. Using principal component analysis, we identified a cluster of exclusively transplanted patients with a median of 23 years of immunosuppression duration, significantly more aggressive biological characteristics, and higher pSer392p53 expression. pSer392p53 was expressed in the whole tumor, suggesting an early carcinogenic event in the course of prolonged immunosuppression. This high, diffuse pSer392p53 expression, corresponding to a high level of DNA damage, might be useful to identify aggressive cutaneous squamous cell carcinomas in kidney-transplant recipients to treat them more aggressively.

## 1. Introduction

**Figure 1:**
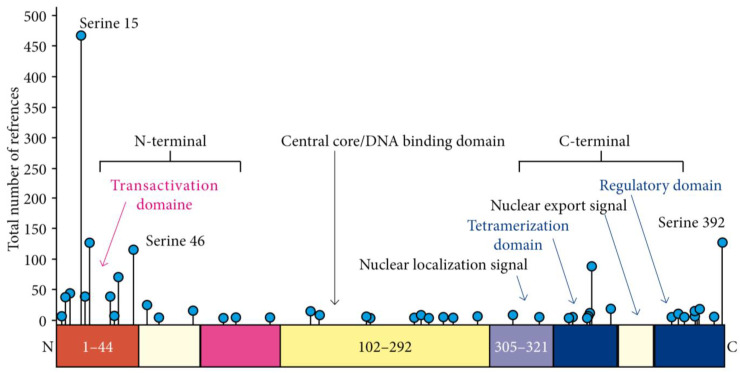
p53 protein structure and the 43 different serine phosphorylation sites.

Cutaneous squamous cell carcinomas (cSCC) are frequent among kidney-transplant recipients. They are usually multiple, with an increasing incidence correlated to immunosuppression duration and a cumulative risk of 40% 20 years after transplantation [1]. The multiple carcinogenesis observed in these patients could be explained by a “field effect” linked to low levels of chronic inflammation with a Th2-dominant microenvironment, a reduced number of natural killer cells, a tolerogenic phenotype of dendritic cells, and an impaired antigen-presenting function to escape immune destruction [2,3]. Exposure to ultraviolet (UV) radiation, particularly on fair skin, induces DNA damage, which activates protective mechanisms, including the p53 pathway [4]. *TP53* tumor suppressor gene inactivation, mainly through mutations, is thus at the cornerstone of UV-induced carcinogenesis [5]. In a small series of kidney-transplant patients with cSCC, the *TP53* mutation prevalence ranged from 36 to 63% [6,7]. In contrast, the prevalence and role of p53 post-translational modifications, including p53 phosphorylation, remain largely unknown. Most phosphorylation events occur early in response to genotoxic stress, resulting in p53 stabilization, accumulation, and increased activity, in turn leading to cell cycle arrest, DNA repair, and cell death apoptosis when necessary [8,9]. To date, 43 sites have been reported to be phosphorylated, mainly in the transactivation N-terminal and sequence-specific DNA-binding C-terminal domains (Figure 1). The latter includes the regulatory domain, which contains Serine 392. In preclinical models of UV-induced carcinogenesis, abolishing Serine 392 (Ser392) phosphorylation increased cancer incidence [10]. In this study, we wanted to investigate the role of Serine 392 phosphorylation in cutaneous SCC.

## 2. Results and Discussion

In our study, we determined the phosphorylated status of Ser392p53 (pSer392p53) in cSCC in 25 French kidney-transplant patients and compared them to 22 cSCC from non-transplanted patients. The median age at diagnosis of the first cSCC was 65 years for transplant patients vs. 77 years for non-transplant patients (*p* = 0.006). In transplanted patients, cSCC occurred after a median period of immunosuppression of 19 years, with a median number of 15 cSCC per patient vs. 1 among non-transplanted patients (*p* < 0.001). The histological characteristics were more aggressive among transplant recipients (significantly higher Clark’s level and peri-neural invasion), with a trend for a higher prevalence of lymph node metastases (*p* = 0.09) (Table 1). 

**Figure 2:**
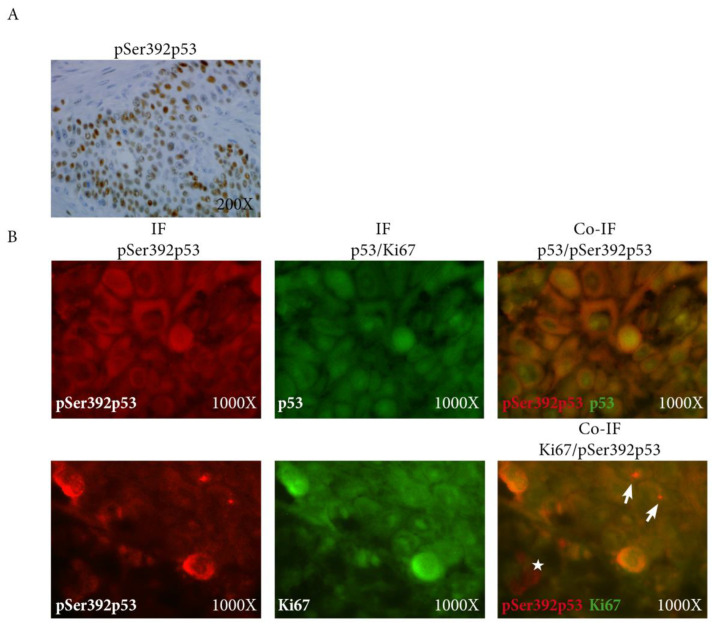
(**A**) Expression of pSer392p53 using immunohistochemistry showing exclusively nuclear coherent with the nuclear export inhibition of the phosphorylated form of p53; (**B**) Co-immuno- fluorescence of pSer392p52 (red) with p53 (green, **upper panel**) and Ki67 (green, **lower panel**) showing the co-localization p53 and pSer392p53 (**upper panel**), and also of pKi67 with pSer392p53 in the invasive front in some cells (arrows) but not all (star) (**lower panel**); IF: immunofluorescence, pSer392p53: Serine 392 phosphorylated p53.

We then assessed Ki67, p53, and pSer392p53 expression using immunohistochemistry. We did not show any significant difference between the two groups (Table 1). Interestingly, pSer392p53 staining was exclusively nuclear (Figure 2A), consistent with the nuclear export inhibition of this phosphorylated form of p53 [11]. 

**Figure 3:**
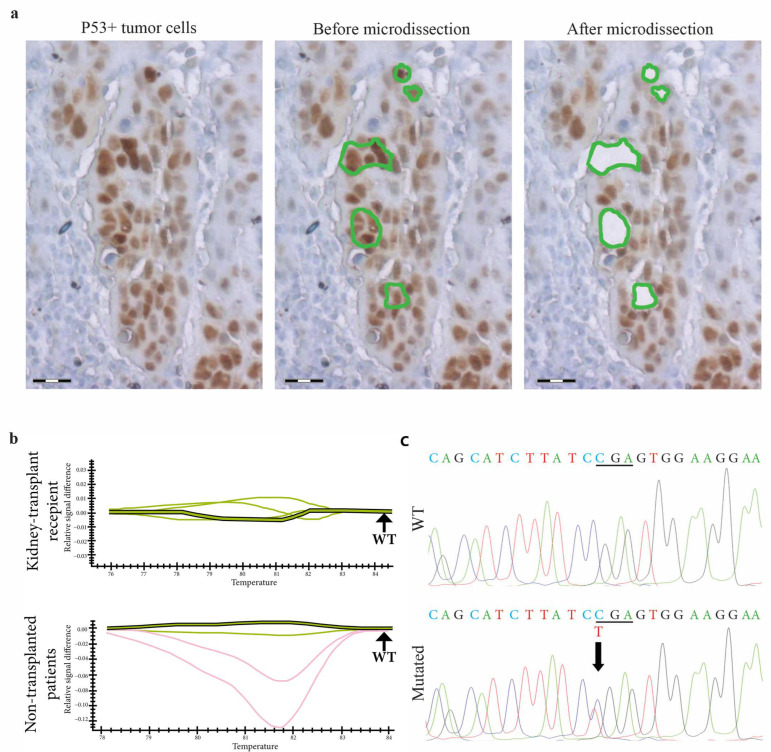
*TP53* gene molecular analysis in p53expressing cells from skin SCCs in kidney-transplant recipients and non-transplanted patients. (**a**) In a skin SCC from a kidney-transplant recipient, p53-expressing tumor cells are laser-microdissected for further molecular analysis. Scale bars = 50 µm. (**b**) *TP53* exon 6A DNA PCR-HRM shows profiles of different samples of skin SCCs from kidney-transplant recipients and non-transplanted patients. Shifted curves (red curves) suggest that *TP53* is mutated in these samples compared to wild-type controls (green curves). (**c**) Using the Sanger method, the sequencing of *TP53* identifies a missense mutation (upper panel), a C to T first base substitution in codon 586 compared to the wild-type sequence (lower panel).

In addition, on laser-microdissected p53-expressing cancer cells (Figure 3), there was no difference between the two groups in terms of mutation prevalence, which was low: 18% vs. 8%, respectively, in non-transplanted vs. transplanted patients (*p* = 0.8) (Table 2). 

When we ran a correlation matrix between quantitative clinical and biological data, a higher Ki67 proliferation index was associated with histological parameters of worse prognosis (Table 3). 

The expression of pSer392p53 was significantly correlated with p53 expression (correlation coefficient 0.83 in the invasive front, *p* < 0.0001), as confirmed on tissue section using double immunofluorescence staining (Figure 2B, upper panel). Interestingly, Ki67 was also significantly associated with pSer392p53 expression (correlation coefficient 0.41 in the invasive front, *p* < 0.05), but not with p53 expression. This was particularly true in the invasive front using double immunofluorescence staining (Figure 2B, lower panel). 

**Figure 4:**
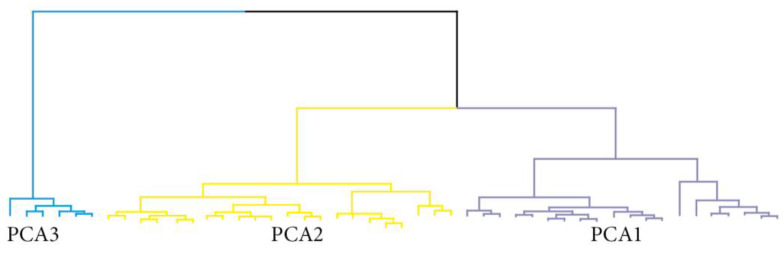
Dendrogram of the hierarchical cluster analysis showing the three clusters identified using principal component analysis.

Using principal component analysis based on clinical and biological markers (Box 1), we identified three clusters (Figure 4, Table 4).

Box 1 **Set of quantitative data used for principal component analysis.**

Age at diagnosis

N° of carcinomas

Thickness

Clark’s level

Ki 67 center and invasion

p53 center and invasion

p53 center and invasion

Phosphorylated p53 center and invasion

**Figure 5:**
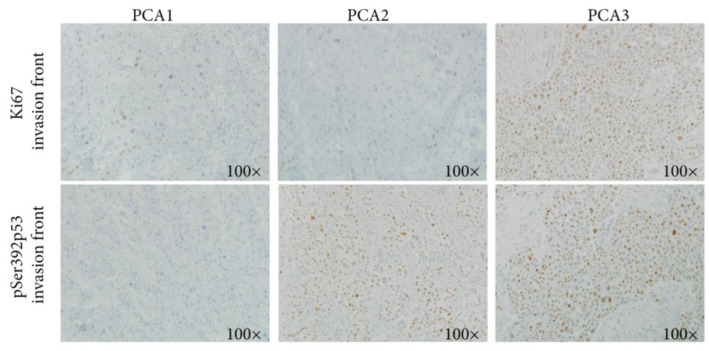
Using immunohistochemistry, a higher Ki67 expression is observed in both the tumor center and the invasive front in the PCA3 cluster, compared to PAC1 and PCA2.

In particular, the PCA3 group included exclusively transplanted patients with a median age of 61.5 years, at least 2 transplantations, and a median of 23 years of immunosuppression duration. They presented significantly more aggressive biological characteristics, with a median number of 24 cSCCs, higher lymph node metastatic potential, and higher Ki67 expression, both in the tumor center and in the invasive front (Figure 5). For pSer392p53 expression, it was also significantly higher in this cluster of patients, and this was particularly marked in the tumor center (Table 4).

Overall, in the PCA3 cluster, pP53 was expressed in the whole tumor and not only in the invasive front, suggesting an early event associated with the carcinogenesis of these forms of cSCCs in the context of prolonged immunosuppression. 

Phosphorylation is the most widely studied post-translational modification of p53, usually associated with its stabilization and accumulation [12]. Phosphorylation of the highly conserved Serine 392 stabilizes the tetramer [13] and inhibits its nuclear export, with enhanced DNA-binding affinity [11]. Furthermore, Serine 392 phosphorylation promotes p53 mitochondrial translocation and transcription-independent apoptosis [8,14]. These functions of pSer392p53 make it a protective factor. 

Here, we identified a subgroup of kidney-transplant patients with prolonged immunosuppression and highly aggressive squamous cell carcinomas expressing pSer392p53. In these patients, the high pSer392p53 expression, corresponding to a high level of p53 activation, could reflect a high level of DNA damage and thus more aggressive disease. 

This can be considered in line of preclinical mouse model with the p53.S389A mutation that showed increased sensitivity to UV-induced damage due to compromised transcriptional activation of p53 target genes and apoptosis. This Serine 392 mutation abolishes p53 phosphorylation, limiting the DNA-binding capacity of p53 [10]. Therefore, in this remarkable clinical situation, high levels of pSer392p53 expression could be considered a marker of intense chronic stress.

The limitations of our study are the small sample size and the fact that all participants were from one hospital, which limits the generalization of the results. Due to the limited number of patients and of several follow-up missing data, we could not perform a survival analysis. 

Recently, new therapies showed very promising results in locally advanced unresectable or metastatic cSCCs. Cetuximab, an anti-Epithelial Growth Factor Receptor (EGFR), achieves a 69% disease control rate [15], and pembrolizumab, an anti-PDL1, leads to a 42% response rate in chemotherapy-pretreated cSCCs [16,17,18]. More recently, cemiplimab, an anti-PD1, showed a 47% response rate as monotherapy in metastatic disease with a 17% complete response and a median duration of response of 41 months, which led to its approval by both the FDA and EMA [19,20,21,22]. For localized SCC, combining cetuximab and radiotherapy after surgery increased progression-free survival compared to each of the treatments as monotherapy [23,24]. In the neoadjuvant setting, cemiplimab was associated with very high pathological complete response rates, up to 70% [25,26], in particular in tumors with high PDL1 expression [25].

## 3. Materials and Methods

### 3.1. Patients and Samples

Between 2004 and 2015, skin SCCs were diagnosed in 25 kidney-transplant recipients at the same university hospital. A gender-matching selection recruited 22 non-transplanted patients diagnosed with sporadic SCC during this period, with no history of any other disease associated with durable immunosuppression. The SCC histological diagnoses were reviewed by two different pathologists (CG, AJ). The last SCC surgical tumor tissue sample for each patient was collected for diagnostic purposes and was formalin-fixed and paraffin-embedded. According to the European consensus-based interdisciplinary guideline, patients with in situ SCC were treated by surgical resection, cryosurgery, superficial skin ablation, or photodynamic therapy. Those with invasive SCC had a comprehensive radiological work-out, and then they were treated by surgical resection, if possible, and complementary radiotherapy when needed. Inoperable cSCCs were treated by radiotherapy combined or not with systemic therapy such as cetuximab [23]. For metastatic disease, the combination of platinum salt with cetuximab was used as a standard of care despite low evidence [27,28,29,30], until the approval of immunotherapy by cemiplimab as it showed better progression-free and overall survival results [19,20,30,31]. The different immunomodulatory treatments as well as follow-up data are detailed in Supplementary Table S1. This work was carried out in accordance with the Code of Ethics of the World Medical Association (Declaration of Helsinki). In compliance with French bioethics law (2004-800; 8 June 2004), all patients had been informed of the research use of the part of their samples remaining after diagnosis had been established, and none opposed it. 

### 3.2. Immunohistochemistry

The detection of p53, phospho-serine392-p53 and Ki67-expressing tumor cells was carried out on 5 µm thick serial paraffin sections using automated indirect immunoperoxidase staining (Benchmark XT; Roche, Tucson, Arizona, United States of America). Mouse anti-human p53 antibody (clone DO7, Dako, Glostrup, Denmark), rabbit anti-human phospho-serine392-p53 antibody (clone EP1889Y, Abcam, Cambridge, UK), and mouse anti-human Ki67 antibody (clone MIB-1, Dako, Glostrup, Denmark) were used as the primary antibodies for immunohistochemical staining. The systematic controls used were the absence of a primary antibody and the use of an irrelevant primary antibody of the same isotype.

All stained slides were assessed independently by three pathologists blinded to diagnosis and clinical data (CG, MB, AJ). p53, phospho-serine392-p53, and Ki67-positive tumor cells were counted on five different fields at ×400 magnification and were representative of different tumor areas. A ProvisAX70 microscope (Olympus, Tokyo, Japan) with a wide-field eyepiece number of 26.5 was used, providing a field size of 0.344 mm^2^ at ×400 magnification. For each field, 100 tumor cells were analyzed. The percentage of expressing cells was defined via a labeling index and determined independently for each marker by three pathologists (CG, MB, AJ). The results were expressed as means ± standard errors of the means. The invasive front of the tumor was defined as the three layers of tumor cells at the front edge between the tumor and the host organ.

### 3.3. Immunofluorescence

An indirect fluorescence method was used on 5 μm thick serial tissue sections to detect the expression of p53, phospho-serine392-p53, and Ki67. Rabbit anti-p53 (phosphoS392) (ab134190, 1:200, Abcam, Cambridge, UK), mouse anti-P53 (MA5-12557, 1/50, Thermo, Waltham, MA, USA), mouse anti-Ki67 (ab238019, 1/100, Abcam, UK) were used as primary antibodies, as were FITC goat anti-rabbit IgG (ab6717, 1/200, Abcam, UK) and Texas red sheep anti-Mouse IgG (ab6806, 1/200, Abcam, UK). The tissue sections were analyzed on a motorized Z-axis microscope (BX-61-Olympus, Tokyo, Japan) using epi-fluorescent light. Microscope images obtained through an UPlan-FI 100x/1.3NA objective were captured using Cell-F-software, version 5.1.2640. 

### 3.4. Laser-Microdissection

For each SCC sample from kidney-transplant recipients and non-transplanted patients, serial 7 µm thick deparaffinized sections were first immunostained with an anti-human p53 antibody. Using a PALM-Microbeam/Zeiss system, laser microdissection was performed on p53+ tumor cells with a pulsed UV-A nitrogen laser (337 nm) used to cut and catapult microdissected cells directly into the buffer-containing cap of a microfuge tube. At least 5000 invasive p53-expressing tumor cells were laser-microdissected for each sample for *TP53* gene analysis. As controls, 5000 p53-negative cells were also laser-microdissected for each sample.

### 3.5. TP53 Gene Screening with High Resolution Melt (HRM)-Polymerase Chain Reaction (PCR) and Sequencing in p53-Expressing Tumor Cells

DNA extraction was performed on laser-microdissected p53-expressing tumor cells using the DNeasy Qiagen Kit^®^ (Qiagen S.A, Courtaboeuf, France). DNA quality was checked by spectrometric assay (NANODROP^®^ ND-1000 spectrophotometer, Thermo Scientific, Wilmington, North California, USA). PCR was carried out on the Bio-Rad CFX96 Real-Time Detection System (Bio-Rad, Hercules, CA, USA) in a total volume of 20 μL containing 5 µL of genomic DNA (20 ng), 15 µL of SsoFastTMEvagreen supermix 1X (Bio-Rad, Hercules, CA, USA) and 0.4 μM of each forward and reverse primer. Each PCR run included a no-template control and a wild-type TP53 control. The PCR was performed with an initial denaturing step at 94 °C for 2 min, followed by 45 cycles of denaturing (95 °C for 5 s) and annealing (60 °C for 10 s). After PCR, a post-amplification melting curve program was initiated by heating to 95 °C for 1 min, cooling to 50 °C for 1 min, and continuously increasing the temperature by 0.2 °C to finally reach 95 °C. Post-amplification fluorescent melting curves were analyzed with Precision Melt Analysis Software, version 1.3 (Bio-Rad, Hercules, CA, USA). Sequencing of the shift fragment for exons 4 to 10 of *TP53* determined by HRM-PCR screening was performed using the Sanger method. Primers were designed from the National Center for Biotechnology Information (NCBI) reference sequence X54156. Amplicons 80–150 bp in length covered the coding sequence and exon-intron boundaries. All forward primers were tailed with the M13-Universal nucleotidic sequence for sequencing standardization. 20 µL of the PCR product were purified using ExoSAP-IT product cleanup (USB Corporation, Cleveland, OH, USA). Labeling was performed using the BigDye^®^ Terminator v1.1 Sequencing Kit (Applied Biosystems, Foster City, CA, USA) in both forward and reverse directions. The reaction was run according to the following protocol: An initial denaturing step at 94 °C for 3 min; 25 cycles at 94 °C for 10 s; annealing temperature at 60 °C for 20 s; BDX-terminator purified products were run on a 16-capillary automated sequencer (ABI-PRISM^®^-3130xl-Genetic-Analyzer, Applied-Biosystems, Foster-City, CA, USA). SeqScape-Software v2.5 (Applied-Biosystems, Foster-City, CA, USA) enabled nucleotide changes to be determined.

### 3.6. Statistical Analysis

Quantitative data were described as medians with the interquartile range (IQR), and qualitative data was described as numbers and proportions (%). 

Patients who had a minimum of one transplantation were first compared to those with no transplantation, using chi2 or Wilcoxon’s test as appropriate. 

We then performed a correlation matrix on quantitative data using Spearman’s coefficient as appropriate for data with a non-normal distribution.

Because there was a strong correlation between the quantitative data and the heterogeneity of our cohort, we ran a hierarchical cluster analysis on principal component analysis (HCP/PCA) to identify homogeneous groups of patients [32]. Hierarchical cluster analysis based on principal component analysis (HCP/PCA) is based on a multivariate combination of a set of variables. Here, we included age, number of carcinomas, thickness, Clark’s level, and biomarker expressions. The selection process for the most informative dimensions of HCP/PCA was based on the eigenvalue. All dimensions with an eigenvalue ≥ 1.0 were considered to select the optimal number of clusters. A scree plot was presented to account for the percentage of explained variance by each dimension and the cumulative explained variance of the dimensions assessed. A dendrogram was plotted to show the optimal number of clusters retained for our study. We performed a multiway comparison across PCA classes using the chi2 test or the Kruskal–Wallis test as appropriate, followed by a linear regression to assess the strength of association between PCA classes (in reference to class 1) and (i) an increase in the number of transplants and (ii) an increase in time since first transplant.

All tests were two-sided, and the threshold for statistical significance was set at *p* < 0.05. The data were analyzed using R statistical software (version 4.1.0, R Foundation for Statistical Computing, Vienna, Austria; http://www.r-project.org, accessed on 23 november 2023).

We could not perform statistical analysis of survival data because of the large amount of censured data, with 78% of patients being lost at follow-up at the time of analysis.

## 4. Conclusions

Typically, pSer392p53 expression could be used to identify poor-prognosis cSCCs, particularly in kidney-transplant recipients. In cases of high pSer392p53 expression, a treatment with cemiplimab could be discussed before surgery, even in the absence of other poor prognostic factors. Such a biomarker should be validated in future clinical trials using new medical therapies. Further series are required to confirm the value of pSer392p53 expression to guide treatment.

## Figures and Tables

**Table 1:** Characteristics of patients and samples.

Variables	N (%) or Median [Q1–Q3]	Transplantation (*n* = 25)	No Transplantation (*n* = 22)	*p* *
**Age at diagnosis** (y), *n* = 47	71.0 ± 18.0	65.0 ± 15.0	77.0 ± 18.0	**0.006**
**Gender** (male), *n* = 47	30 (64)	16 (64)	14 (64)	0.98
**Transplantation**				
Yes	25 (53)	-	-	-
Number	-	2.0 [1.0–2.0]	-	-
Time since first transplant (y)	-	19.0 [5.5–26.0]	-	-
**Number of carcinomas**, *n* = 47	3.0 ± 17.0	15.0 ± 18.0	1.0 ± 0.75	**<0.001**
**Thickness** (mm), *n* = 42	4.0 ± 7.1	4.5 ± 12.0	3.2 ± 3.5	0.32
**Clark’s level** ≥ 4 (yes), *n* = 47	40 (85)	22 (88)	18 (82)	0.19
**Level of differentiation**, *n* = 47				0.22
Good	41 (87)	22 (88)	19 (86)	
Fair	4 (8.5)	1 (4)	3 (14)	
Poor	2 (4.5)	2 (8)	0 (0)	
**Peri-neural invasion** (yes), *n* = 45	5 (11)	5 (20)	0 (0)	**0.02**
Metastases				
Lymph node (yes), *n* = 42	4 (10)	4 (16)	0 (0)	0.09
Visceral (yes), *n* = 41	3 (7)	3 (12)	0 (0)	0.15
**Biomarker expression** (%)				
Ki67 center, *n* = 42	0.0 ± 10.0	2.5 ± 45.0	0.0 ± 6.0	0.21
Ki67 invasion, 42	20.0 ± 47.5	12.5 ± 65.0	20.0 ± 22.5	0.95
p53 center, *n* = 45	12.5 ± 47.5	30.0 ± 50.0	5.0 ± 20.0	0.08
p53 invasion, *n* = 42	70.0 ± 65.0	75.0 ± 62.5	50.0 ± 65.0	0.69
pSer392 p53 center, *n* = 41	0.0 ± 20.0	0.0 ± 30.0	0.0 ± 6.0	0.16
pSer392p53 invasion, *n* = 44	27.5 ± 70.0	37.5 ± 80.0	10.0 ± 70.0	0.45

pSer392p53: phosphorylated Serine 392 p53. * *p*-value for Chi2 or Wilcoxon’s test as appropriate.

**Table 2:** PCR-HRM and Sanger sequencing profile comparison of kidney-transplant recipients and non-transplanted patients.

	Kidney-Transplant Recipients			Non-Transplanted Patients		
Sample	PCR-HRM	Sanger Sequencing	Sample	PCR-HRM	Sanger Sequencing	
1	Exon 5B	p.V157F c.469 G > T	1	Exon 5B	p.V157F c.469 G > T	
2	Exon 7B	p.R248W c.742 C > T	2	Exon 6A	p.R196 * c.586 C > T	
3	Exon 9B	WT	3	Exon 6B	p.E221V c.662 A > T	
4	Exon 7A	WT	4	Exon 8A	p.R282W c.844 C > T	
5	Exon 6A	WT	5	Exon 5B	WT	
6	Exon 4A	WT	6	Exon 6B	WT	
7	Exon 8A	WT	7	Exon 6B	WT	
8	Exon 6B	WT	8	Exon 6B	WT	
9	Exon 6A	WT	9	Exon 7A	WT	
10	Exon 6A	WT	10	WT	-	
11	Exon 5C	WT	11	WT	-	
12	Exon 9A	WT	12	WT	-	
13	WT	-	13	Insufficient material		
14	WT	-	14	Insufficient material		
15	WT	-	15	Insufficient material		
16	WT	-	16	Insufficient material		
17	WT	-	17	WT	-	
18	WT	-	18	WT	-	
19	WT	-	19	WT	-	
20	WT	-	20	WT	-	
21	WT	-	21	WT	-	
22	WT	-	22	WT	-	
23	WT	-				
24	WT	-				
25	WT	-				

PCR-HRM: polymerase chain reaction-heat resolution melting; WT: wild type.

**Table 3:** Correlation matrix between quantitative variables.

Variables	Age	N° Carcinomas	Thickness	Clark’s Level	Ki67 Center	Ki67 Invasion	P53 Center	P53 Invasion	pSer392p53 Center	pSer392p53 Invasion
**Age**	**1.00**									
**N° carcinomas**	−0.47 **	**1.00**								
**Thickness**	**−0.31**	0.26	**1.00**							
**Clark’s level**	−0.29	**0.31**	0.42 *	**1.00**						
**Ki67 center**	−0.26	0.29	0.36 *	0.44 *	**1.00**					
**Ki67 invasion**	−0.10	0.16	**0.32**	0.36 *	0.85 ***	**1.00**				
**p53 center**	−0.07	0.28	0.03	0.17	0.30	0.27	**1.00**			
**p53 invasion**	0.06	0.19	−0.19	−0.001	0.02	0.14	0.69 ***	**1.00**		
**pSer392p53 center**	−0.23	**0.34**	0.23	0.25	0.39 *	0.42 *	0.74 ***	0.59 ***	**1.00**	
**pSer392p53 invasion**	−0.05	0.30	0.03	0.13	0.26	0.41 *	0.53 **	0.83 ***	0.69 ***	**1.00**

Bold: significant results with a threshold of >30%. * < 0.05, ** < 0.01, *** < 0.0001.

**Table 4:** Phenotype associated with the PCA’s classes.

Variables	N (%) or Median ± IQR			p *
	PCA1 (*n* = 20)	PCA2 (*n* = 21)	PCA3 (*n* = 6)	
**Age at diagnosis** (y)	65.0 ± 15.5	75.0 ± 12.0	**61.5 ± 6.0**	**0.009**
**Gender** (male)	13 (65)	12 (54.5)	5 (83)	0.49
**Transplantation**				
Yes	10.0 (50)	9 (41)	**6 (100)**	**0.03**
**N° of transplants**	0.5 ± 2.0	0.0 ± 1.0	**2.0 ± 1.0**	**0.01**
Time since first transplant (y)	0.0 ± 14.5	0.0 ± 2.0	**23.0 ± 8.0**	**0.007**
**N° of carcinomas**	3.0 ± 13.5	2.0 ± 6.0	**24 ± 17.0**	**0.02**
**Thickness** (mm)	4.0 ± 9.5	3.1 ± 4.0	7.5 ± 9.5	0.15
**Clark’s level** ≥ 4 (yes)	15 (75)	19 (86)	6 (100)	0.20
**Level of differentiation**				0.89
Good	18 (90)	18 (86)	5 (83)	
Fair	1 (5)	2 (9.5)	1 (7)	
Poor	1 (5)	1 (4.5)	0 (0)	
**Peri-neural invasion** (yes)	4 (20)	0 (0)	1 (17)	0.07
**Metastases**				
Lymph node (yes)	2 (10)	0 (0)	**2 (33)**	**0.05**
Visceral (yes)	2 (10)	1 (4.5)	0 (0)	0.59
**Biomarker expression** (%)				
Ki67 center	0.0 ± 9.0	0.0 ± 5.0	**70.0 ± 22.5**	**0.0001**
Ki67 invasion	20.0 ± 30.0	10.0 ± 27.0	**75.0 ± 10.0**	**0.0004**
p53 center	0.0 ± 0.0	25.0 ± 40.0	**60.0 ± 27.5**	**<0.0001**
p53 invasion	20.0 ± 26.0	90.0 ± 10.0	**90.0 ± 7.5**	**0.04**
pSer392-p53 center	0.0 ± 0.0	5.0 ± 30.0	**50.0 ± 22.5**	**<0.0001**
pSer392-p53 invasion	0.0 ± 9.0	70.0 ± 57.5	**75.0 ± 17.5**	**<0.0001**

* *p*-value for chi2 or Kruskal–Wallis test as appropriate. Bold: significant *p*-value at the threshold of 5%.

## Data Availability

The datasets used and/or analyzed in the current study are available from the corresponding author upon reasonable request.

## Supplementary Materials

The following supporting information can be downloaded at: https://www.mdpi.com/article/10.3390/ijms25021147/s1.

## References

[1] Euvrard S., Kanitakis J., Claudy A. (2003). Skin cancers after organ transplantation. N. Engl. J. Med..

[2] Hofbauer G.F., Bouwes Bavinck J.N., Euvrard S. (2010). Organ transplantation and skin cancer: Basic problems and new perspectives. Exp. Dermatol..

[3] Carroll R.P., Segundo D.S., Hollowood K., Marafioti T., Clark T.G., Harden P.N., Wood K.J. (2010). Immune phenotype predicts risk for posttransplantation squamous cell carcinoma. J. Am. Soc. Nephrol..

[4] Yarosh D.B., Boumakis S., Brown A.B., Canning M.T., Galvin J.W., Both D.M., Kraus E., O’Connor A., Brown D.A. (2002). Measurement of UVB-Induced DNA damage and its consequences in models of immunosuppression. Methods.

[5] Laing M.E., Kay E., Conlon P., Murphy G.M. (2007). Genetic factors associated with skin cancer in renal transplant patients. Photodermatol. Photoimmunol. Photomed..

[6] Queille S., Luron L., Spatz A., Avril M.F., Ribrag V., Duvillard P., Hiesse C., Sarasin A., Armand J.P., Daya-Grosjean L. (2007). Analysis of skin cancer risk factors in immunosuppressed renal transplant patients shows high levels of UV-specific tandem CC to TT mutations of the *p53* gene. Carcinogenesis.

[7] McGregor J.M., Harwood C.A., Brooks L., Fisher S.A., Kelly D.A., O’Nions J., Young A.R., Surentheran T., Breuer J., Millard T.P. (2002). Relationship between *p53* codon 72 polymorphism and susceptibility to sunburn and skin cancer. J. Investig. Dermatol..

[8] Bode A.M., Dong Z. (2004). Post-translational modification of p53 in tumorigenesis. Nat. Rev. Cancer.

[9] Gu B., Zhu W.G. (2012). Surf the post-translational modification network of p53 regulation. Int. J. Biol. Sci..

[10] Bruins W., Zwart E., Attardi L.D., Iwakuma T., Hoogervorst E.M., Beems R.B., Miranda B., van Oostrom C.T., van den Berg J., van den Aardweg G.J. (2004). Increased sensitivity to UV radiation in mice with a p53 point mutation at Ser389. Mol. Cell. Biol..

[11] Kim Y.Y., Park B.J., Kim D.J., Kim W.H., Kim S., Oh K.S., Lim J.Y., Kim J., Park C., Park S.I. (2004). Modification of serine 392 is a critical event in the regulation of p53 nuclear export and stability. FEBS Lett..

[12] Minamoto T., Buschmann T., Habelhah H., Matusevich E., Tahara H., Boerresen-Dale A.L., Harris C., Sidransky D., Ronai Z. (2001). Distinct pattern of p53 phosphorylation in human tumors. Oncogene.

[13] Sakaguchi K., Sakamoto H., Lewis M.S., Anderson C.W., Erickson J.W., Appella E., Xie D. (1997). Phosphorylation of serine 392 stabilizes the tetramer formation of tumor suppressor protein p53. Biochemistry.

[14] Castrogiovanni C., Waterschoot B., De Backer O., Dumont P. (2018). Serine 392 phosphorylation modulates p53 mitochondrial translocation and transcription-independent apoptosis. Cell Death Differ..

[15] Maubec E., Petrow P., Scheer-Senyarich I., Duvillard P., Lacroix L., Gelly J., Certain A., Duval X., Crickx B., Buffard V. (2011). Phase II study of cetuximab as first-line single-drug therapy in patients with unresectable squamous cell carcinoma of the skin. J. Clin. Oncol..

[16] Maubec E., Boubaya M., Petrow P., Beylot-Barry M., Basset-Seguin N., Deschamps L., Grob J.J., Dreno B., Scheer-Senyarich I., Bloch-Queyrat C. (2020). Phase II study of pembrolizumab as first-line, single-drug therapy for patients with unresectable cutaneous squamous cell carcinomas. J. Clin. Oncol..

[17] Grob J.J., Gonzalez R., Basset-Seguin N., Vornicova O., Schachter J., Joshi A., Meyer N., Grange F., Piulats J.M., Bauman J.R. (2020). Pembrolizumab Monotherapy for Recurrent or Metastatic Cutaneous Squamous Cell Carcinoma: A Single-Arm Phase II Trial (KEYNOTE-629). J. Clin. Oncol..

[18] Hughes B.G.M., Munoz-Couselo E., Mortier L., Bratland A., Gutzmer R., Roshdy O., Gonzalez Mendoza R., Schachter J., Arance A., Grange F. (2021). Pembrolizumab for locally advanced and recurrent/metastatic cutaneous squamous cell carcinoma (KEYNOTE-629 study): An open-label, nonrandomized, multicenter, phase II trial. Ann. Oncol..

[19] Hughes B.G., Grob J.-J., Bowyer S.E., Day F., Ladwa R., Stein B., Couselo E.M., Basset-Seguin N., Guminski A., Mortier L. (2022). 818P Phase II confirmatory study of cemiplimab (350mg IV Q3W) in patients with locally advanced or metastatic cutaneous squamous cell carcinoma (CSCC): Study 1540 Group 6. Ann. Oncol..

[20] Migden M., Schmults C., Khushanlani N., Guminski A., Chang A., Lewis K., Ansstas G., Bowyer S., Hughes B., Schadendorf D. (2022). 814P Phase II study of cemiplimab in patients with advanced cutaneous squamous cell carcinoma (CSCC): Final analysis from EMPOWER-CSCC-1 groups 1, 2 and 3. Ann. Oncol..

[21] Migden M.R., Rischin D., Schmults C.D., Guminski A., Hauschild A., Lewis K.D., Chung C.H., Hernandez-Aya L., Lim A.M., Chang A.L.S. (2018). PD-1 Blockade with Cemiplimab in Advanced Cutaneous Squamous-Cell Carcinoma. N. Engl. J. Med..

[22] Migden M.R., Khushalani N.I., Chang A.L.S., Lewis K.D., Schmults C.D., Hernandez-Aya L., Meier F., Schadendorf D., Guminski A., Hauschild A. (2020). Cemiplimab in locally advanced cutaneous squamous cell carcinoma: Results from an open-label, phase 2, single-arm trial. Lancet Oncol..

[23] Palmer J.D., Schneider C.J., Hockstein N., Hanlon A.L., Silberg J., Strasser J., Mauer E.A., Dzeda M., Witt R., Raben A. (2018). Combination of post-operative radiotherapy and cetuximab for high-risk cutaneous squamous cell cancer of the head and neck: A propensity score analysis. Oral Oncol..

[24] Kreinbrink P.J., Mierzwa M.L., Huth B., Redmond K.P., Wise-Draper T.M., Casper K., Li J., Takiar V. (2021). Adjuvant radiation and cetuximab improves local control in head and neck cutaneous squamous cell carcinoma: Phase II study. Head Neck.

[25] Gross N.D., Miller D.M., Khushalani N.I., Divi V., Ruiz E.S., Lipson E.J., Meier F., Su Y.B., Swiecicki P.L., Atlas J. (2022). Neoadjuvant Cemiplimab for Stage II to IV Cutaneous Squamous-Cell Carcinoma. N. Engl. J. Med..

[26] Ferrarotto R., Amit M., Nagarajan P., Rubin M.L., Yuan Y., Bell D., El-Naggar A.K., Johnson J.M., Morrison W.H., Rosenthal D.I. (2021). Pilot Phase II Trial of Neoadjuvant Immunotherapy in Locoregionally Advanced, Resectable Cutaneous Squamous Cell Carcinoma of the Head and Neck. Clin. Cancer Res..

[27] Jarkowski A., Hare R., Loud P., Skitzki J.J., Kane J.M., May K.S., Zeitouni N.C., Nestico J., Vona K.L., Groman A. (2016). Systemic Therapy in Advanced Cutaneous Squamous Cell Carcinoma (CSCC): The Roswell Park Experience and a Review of the Literature. Am. J. Clin. Oncol..

[28] Trodello C., Pepper J.P., Wong M., Wysong A. (2017). Cisplatin and Cetuximab Treatment for Metastatic Cutaneous Squamous Cell Carcinoma: A Systematic Review. Dermatol. Surg..

[29] Preneau S., Rio E., Brocard A., Peuvrel L., Nguyen J.M., Quereux G., Dreno B. (2014). Efficacy of cetuximab in the treatment of squamous cell carcinoma. J. Dermatol. Treat..

[30] Stratigos A.J., Garbe C., Dessinioti C., Lebbe C., van Akkooi A., Bataille V., Bastholt L., Dreno B., Dummer R., Fargnoli M.C. (2023). European consensus-based interdisciplinary guideline for invasive cutaneous squamous cell carcinoma: Part 2. Treatment-Update 2023. Eur. J. Cancer.

[31] Petzold A., Steeb T., Wessely A., Schatton T., Berking C., Heppt M.V. (2022). Comparative efficacy analysis identifies immune checkpoint blockade as a new survival benchmark in advanced cutaneous squamous cell carcinoma. Eur. J. Cancer.

[32] Cova T.F., Pereira J.L., Pais A.A. (2013). Is standard multivariate analysis sufficient in clinical and epidemiological studies?. J. Biomed. Inform..

