# Supplementary material for: A Sub-Group of Kidney-Transplant Recipients with Highly Aggressive Squamous Cell Carcinoma Expressing Phosphorylated Serine392p53
Source: Int J Mol Sci. 2024 Jan 17;25(2):1147. doi: 10.3390/ijms25021147 (PMC10816400; doi:10.3390/ijms25021147)
Supplement: Supplementary file 1 [file ijms-25-01147-s001.zip › ijms-2771583-supplementary.pdf]

**Supplementary Table S1.** Data follow-up and immunosuppression regimens of patients.

| Clusters of patients | Patient number | Type of immunosuppression treatement | Follow-up data |
|---|---|---|---|
| PCA1 | P1 | Azathioprine<br>Corticosteroids | Non-specific death |
| | P2 | Corticosteroids<br>Mycophenolic acid<br>Sirolimus | Lost of follow-up |
| | P3 | Non | Specific death |
| | P4 | Everolimus<br>Tacrolimus<br>Corticosteroids | Specific death |
| | P4 | Corticosteroids<br>Tacrolimus | Lost of follow-up |
| | P5 | Corticosteroids<br>Everolimus | Non-specific death |
| | P6 | - | Lost of follow-up |
| | P7 | - | Lost of follow-up |
| | P8 | Corticosteroids<br>Azathioprine<br>Tacrolimus | Lost of follow-up |
| | P9 | Tacrolimus<br>Corticosteroids | Specific death |
| | P10 | - | Lost of follow-up |
| | P11 | - | Non-specific death |
| | P12 | - | Lost of follow-up |
| | P13 | - | Lost of follow-up |
| | P14 | - | Lost of follow-up |
| | P15 | - | Lost of follow-up |
| | P16 | - | Lost of follow-up |
| | P17 | - | Lost of follow-up |
| | P18 | - | Lost of follow-up |
| | P19 | - | Lost of follow-up |
| PCA2 | P20 | Mycophenolic acid<br>Tacrolimus<br>Corticosteroids | Lost of follow-up |
| | P21 | - | Lost of follow-up |
| | P22 | - | Lost of follow-up |
| | P23 | Mycophenolic acid<br>Corticosteroids<br>Tacrolimus | |
| | P24 | Ciclosporine<br>Azathioprine | |
| | P25 | Corticosteroids | Lost of follow-up |
| | P26 | - | Lost of follow-up |
| | P27 | Corticosteroids<br>ciclosporine<br>Tacrolimus<br>Azathioprine | Specific death |

| | | | |
|---|---|---|---|
| | | Mycophenolic acid | |
| | | Everolimus | |
| | P28 | Azathioprine | Lost of follow-up |
| | | Tacrolimus | |
| | P29 | - | Lost of follow-up |
| | P30 | - | Lost of follow-up |
| | P31 | - | Lost of follow-up |
| | P32 | - | Lost of follow-up |
| | P33 | - | Lost of follow-up |
| | P34 | - | Lost of follow-up |
| | P35 | - | Lost of follow-up |
| | P36 | - | Lost of follow-up |
| | P37 | - | Lost of follow-up |
| | P38 | - | Lost of follow-up |
| | P39 | - | Lost of follow-up |
| | P40 | - | Lost of follow-up |
| | P41 | - | Lost of follow-up |
| PCA3 | P42 | Sirolimus | Specific death |
| | | Mycophenolic acid | |
| | P43 | Tacrolimus | Non-specific death |
| | | Mycophenolic acid | |
| | P44 | Tacrolimus | Lost to follow-up |
| | | Mycophenolic acid | |
| | P45 | Corticosteroids | Lost of follow-up |
| | | Tacrolimus | |
| | P46 | Mycophenolic acid | Lost of follow-up |
| | | Tacrolimus | |
| | | Corticosteroids | |
| | P47 | Corticosteroids | Lost of follow-up |
| | | Sirolimus | |

P: Patient.
